# Efficacy of acupuncture on cognitive function in poststroke depression: study protocol for a randomized, placebo-controlled trial

**DOI:** 10.1186/s13063-022-06011-7

**Published:** 2022-01-28

**Authors:** Ling Chen, Yi Chen, Lihua Wu, Wen Fu, Luanmian Wu, Wenbin Fu

**Affiliations:** 1 Second Clinical College, Guangzhou University of Chinese Medicine, Guangzhou, China; 2 Guangzhou University of Chinese Medicine, Guangzhou, China; 3 Shenzhen Bao’an Research Center for Acupuncture and Moxibustion, Guangdong Province, Shenzhen, China; 4 Sanming Project of Medicine in Shenzhen, Guangdong Province, Shenzhen, China

**Keywords:** Poststroke depression, Acupuncture, Efficacy, Cognitive function, Randomized controlled trial, Clinical trial

## Abstract

**Introduction:**

Poststroke depression (PSD) is the most common mental complication after stroke and has a serious impact on functional outcomes and quality of life. Antidepressants are the first-line treatment for PSD, but many reported side effects remain. Clinical research has shown that acupuncture has a positive effect on PSD. This trial aims to study the efficacy and safety of acupuncture for PSD and to explore its effect on cognitive function. It is hypothesized that acupuncture treatment improves depressive symptoms, cognitive behavior, and negative emotion processing bias in PSD.

**Methods:**

In this randomized, placebo-controlled, single-blinded trial, fifty-six people with PSD will be randomly allocated into the intervention (*n*=28) or control (*n*=28) groups. The intervention group will receive acupuncture treatment, and the control group will receive sham acupuncture treatment, in 20 sessions over 4 weeks. The primary outcome is the change from baseline in the Hamilton Depression Scale-17 (HAMD-17) scores at week 4. Secondary outcomes include the Wisconsin Card Sorting Test (WCST) and latency and amplitude of P1, N170, and P3 of the event-related potentials (ERPs) components to assess the changes in cognitive function and electroencephalography. Outcomes are assessed at baseline and post intervention.

**Discussion:**

Acupuncture therapy could become an alternative treatment for PSD, and it is expected that this trial will provide reliable clinical evidence for the future use of acupuncture for the treatment of PSD.

**Trial registration:**

Chinese Clinical Trial Registry ChiCTR1900026948. Registered on 27 October 2019.

**Supplementary Information:**

The online version contains supplementary material available at 10.1186/s13063-022-06011-7.

## Introduction

Stroke is one of the most devastating diseases affecting the aging population in the world and is the main cause of death and disability [1]. Poststroke depression (PSD) is the most frequent emotional disorder resulting from stroke and is characterized by hopelessness and loss of interest and pleasure beyond stroke symptoms. Approximately 33% of stroke survivors suffer from depression [2], which is 4 to 10 times the incidence observed in the general population [3]. PSD not only increases the risk of stroke recurrence [4], but also affects functional outcomes and quality of life [5]. Moreover, the mortality rate in stroke survivors with PSD is 3 to 4 times higher than that in stroke survivors without depression [6]. Although stroke is the direct cause of PSD, the underlying mechanisms of PSD remain unclear. At present, the diagnosis of PSD is mainly based on clinical manifestations combined with scale assessments, as there is a lack of physiological indicators. As approximately one-third of stroke patients experience aphasia [7], 70% of stroke survivors have cognitive impairment [8]. It is difficult to know whether emotion and interest have changed, which poses a great challenge in the treatment of PSD.

Pharmacotherapy is the first-line treatment of PSD. However, antidepressants are ineffective in approximately 40% to 60% of patients [9], and 30% of patients develop refractory depression due to ineffective treatment with multiple antidepressants [9]. In addition, several antidepressants increased the risk of cardiovascular disease and stroke [10], resulting in a low treatment compliance rate. Therefore, there is an urgent need for therapy that is effective, safe, and easy to promote.

Acupuncture is recommended as a promising nonpharmaceutical therapy for major depressive disorder (MDD). Published literature [11] indicates that acupuncture could improve the symptoms of depression evidenced by reductions in Hamilton Depression Scale (HAMD) scores in individuals with PSD. An fMRI study demonstrated that acupuncture may have an antidepressant effect by regulating emotion-related brain areas such as the amygdala and anterior cingulate cortex [12]. Several meta-analyses and systematic reviews have shown that acupuncture may be superior to antidepressants in alleviating the depressive symptoms of PSD [11, 13]. However, these trials did not consider placebo or sham acupuncture treatment to exclude the psychological effects of acupuncture.

Studies have shown that depression is associated with cognitive control deficits with regard to emotional information [14]. MDD is characterized by abnormal cognitive processing of negative bias, which is manifested by paying excessive attention to negative emotional information [15, 16]. The cognitive processing bias of negative emotions penetrates the whole process of depression and is at the core of the development of depressive symptoms. It is very important to understand the effects of acupuncture on cognition as it relates to PSD because understanding the relationship between emotions and cognitive function can help to explain the antidepressant effects. Event-related potentials (ERPs) reflect neural activities in the cognitive process, and because their assessment is noninvasive and has high time resolution (milliseconds), ERP measurements have become the preferred technology for studying neurocognition. According to a recent study, the recurrence and severity of depression may disrupt emotional processing, and the ERP component may be a potential biomarker to predict depression and cognitive function [17]. Thus, ERP can be adopted to explore the effect of acupuncture treatment on the negative emotion processing bias in PSD.

This randomized, placebo-controlled, single-blinded study was designed to explore the efficacy and safety of acupuncture on PSD and to explore its effect on cognitive function. It is hypothesized that acupuncture treatment will improve symptoms of depression, cognitive function, and abnormal negative emotion processing bias in individuals with PSD.

## Methods

### Study setting

**Fig. 1:**
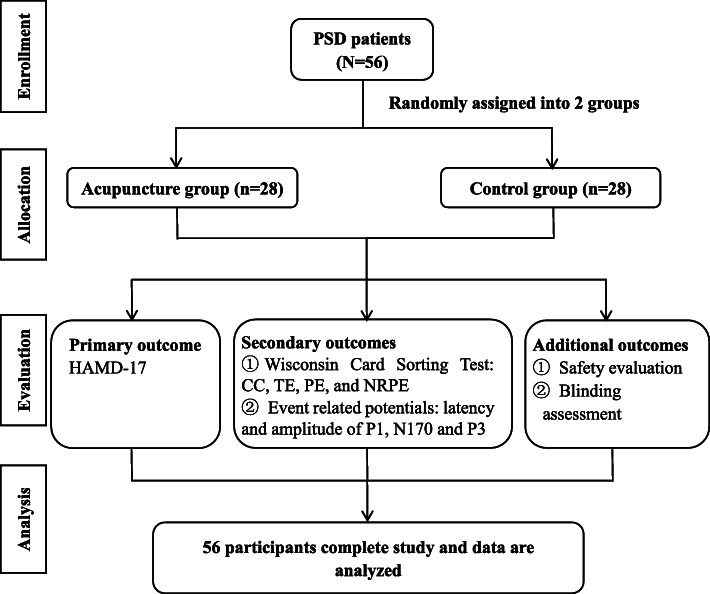
Flow diagram of the study procedure. CC, categories completed; TE, total errors; PE, perseverative errors; NRPE, nonperseverative errors

**Fig. 2:**
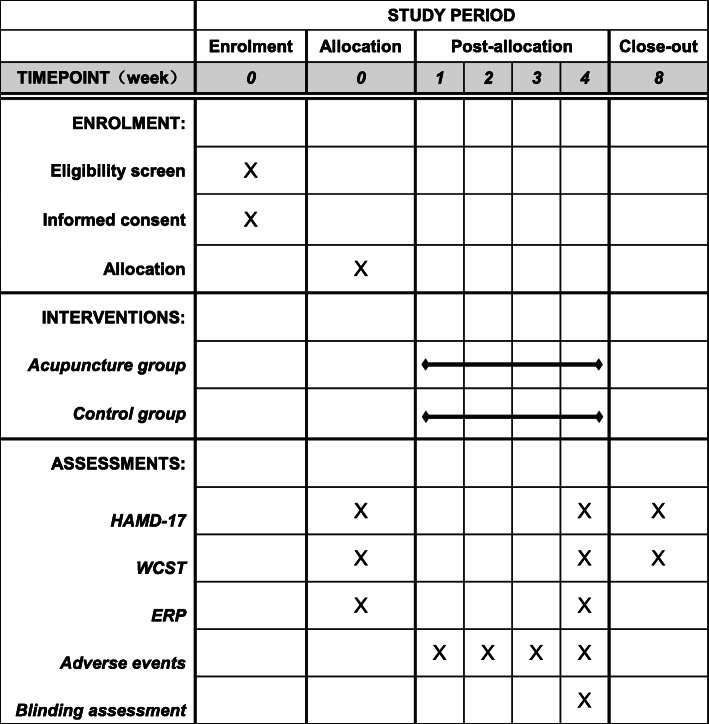
Standard Protocol Items: Recommendations for Interventional Trials (SPIRIT) schedule of enrolment, interventions, and assessments. HAMD, Hamilton Depression Scale; WCST, Wisconsin Card Sorting Test; ERP, Event-related potential

This randomized, placebo-controlled, single-blinded trial will recruit 56 stroke survivors with depression. The framework of the trial is superiority. This study will be conducted at the Department of Acupuncture of The Secondary Medical College of Guangzhou University of Traditional Chinese Medicine, China. The study protocol was registered at the Chinese Clinical Trial Registry (ChiCTR1900026948). The study will be performed in line with the Declaration of Helsinki formulated by the World Medical Association. Written informed consent will be obtained from all participants. All routine interventions will be performed by hospital acupuncture staff. Figure 1 shows the CONSORT flowchart of the trial. This protocol was organized based on the Standard Protocol Items: Recommendations for Interventional Trials (SPIRIT) Checklist (Additional file 1) [18]. Figure 2 shows the SPIRIT outline for enrollment, interventions, and assessments.

### Eligibility criteria

#### Inclusion criteria

The inclusion criteria are as follows: (1) patient meets the criteria for “stroke” and “depression” based on the ICD-10 and are diagnosed with PSD by a psychiatrist; (2) the patient is aged between 40 and 75 years old; (3) ischemic stroke was confirmed by computed tomography (CT) or magnetic resonance imaging (MRI); (4) patient shows syndromes of liver stagnation based on traditional Chinese medicine (TCM) diagnostic criteria; (5) patient has a HAMD-17 score ≥17; and (6) the duration of PSD is less than 6 months. For the TCM diagnostic criteria, we refer to “Expert Consensus of Diagnosis and Treatment of Major Depression with Integrated TCM and Western Medicine” [19].

#### Exclusion criteria

The exclusion criteria are as follows: (1) massive cerebral infarction; (2) suicidal tendency as evaluated by psychiatrist; (3) serious medical diseases, such as brain injury, epilepsy, malignant tumor, congestive heart failure, respiratory failure, renal failure and atrial fibrillation; (4) previous head trauma history, such as coma, vomiting, amnesia, convulsions or medical emergency after injury; (5) severe cognitive impairment (Mini-Mental State Examination ≤21 [20]); (6) schizophrenia, anxiety or other mental disorders; (7) a DSM-IV history of substance abuse or dependence in the 6 months prior to assessment; and (8) metal implants in the body, such as pacemaker and deep brain stimulator.

#### Termination criteria

The termination criteria are as follows: (1) serious adverse reactions or aggravation occurred during the study, and it is not advisable to continue participating in the study; (2) those who withdraw during the study; and (3) those who do not follow the treatment for personal reasons or reject to continue the study.

### Intervention

The enrolled PSD participants receive basic treatment for stroke: basic pharmacotherapy treatment for ischemic stroke and other diseases (such as hypertension, coronary heart disease, and diabetes), and physical therapy and occupational therapy for motor dysfunction will occur every day over 60 min (including preparation). Participants cannot receive antidepressant medication, or on any other acupuncture treatments that may affect the evaluation of the efficacy of the interventions of the study. The participants’ psychological condition will be closely monitored by a psychiatrist throughout the study period. Participants would be withdrawn from the study should any sign of deterioration in mental health condition and will be treated by a psychologist. The acupuncture group will receive acupuncture treatment, and the control group will receive sham acupuncture treatment. The participating physicians in the trial are acupuncture doctors at the Secondary Medical College of Guangzhou University of Traditional Chinese Medicine. As the acupuncturists are responsible for the acupuncture treatment, they are all highly qualified, have a master’s degree in acupuncture and moxibustion, have undergone training in a unified standardized operation plan. Acupuncture treatment will be administered in the supine position, and the temperature of the treatment room is maintained at 26 °C. Participants will receive 20 treatment sessions over a 4-week period at a frequency of 5 times per week.

#### Acupuncture group

Participants in the acupuncture group will receive acupuncture at Baihui (GV20), Yingtang (GV29), Hegu (LI4, bilateral), and Taichong (LR3, bilateral). Disposable stainless-steel needles (0.3 × 40 mm; Suzhou Tianxie Acupuncture Instruments Co., Ltd., Suzhou City, China) will be used. After skin disinfection, sterile adhesive pads will be placed on the acupoints. For GV20 and GV29, acupuncture needles will be inserted through the adhesive pads for approximately 15 mm at an angle of 15°. For bilateral LI4 and LR3, the needles will be inserted vertically to a depth of 20 mm. Following needle insertion, the acupuncturists will twirl the needle handles back and forth to achieve the sensation of achiness, heaviness, and numbness (known as de qi) at all acupoints. The needles will be removed after 20 min.

#### Control group

Participants in the control group will receive sham acupuncture at the same acupoints. In the sham acupuncture group, placebo needles with a blunt tip (0.3 × 40 mm; Suzhou Tianxie Acupuncture Instruments Co., Ltd., Suzhou City, China) and sterile adhesive pads will be used. Those non-invasive placebo devices have been applied in published randomized controlled trials (RCTs) to minimize the physiological effects of skin penetratio n[21]. Furthermore, it was suggested that when applying these placebo devices as control, even acupuncture-experienced participants could be blinded when the application was in a less sensitive poin t[22]. After skin disinfection, sterile adhesive pads will be placed on the acupoints. Then, placebo needles are needled through the pads pressed against the skin without penetration. The procedures and other treatment parameters will be the same as those in the acupuncture group, e.g., the needles will be left in place for 20 min, but there will be no acupuncture needle manipulation.

### Follow-up

To evaluate the persistence of the effects, a follow-up assessment will occur 1 month after the end of the 4-week treatment.

### Outcome measures

The endpoints will be evaluated at baseline (before intervention), week 4 (immediately after intervention), and week 8 (1-month follow-up). We include two figures to show the participant timeline more clearly (see Figs. 1 and 2).

#### Primary outcome

The primary outcome is the change in the degree of depression as measured by the HAMD-17 [23] at week 4. The HAMD-17 consists of a total of 13 items with a total score that can range from 0 to 24, where 0 defines least depressed and 24 describes most depressed.

#### Secondary outcomes

The secondary outcomes include the Wisconsin Card Sorting Test (WCST) and the latency and amplitude of the ERP components P1, N170, and P3 at week 4.

- The WCST [24] is being adopted to assess cognitive function. Participants are required to match response cards to the four stimulus cards along one of the three dimensions (color, form, or number) by pressing one of the 1 to 4 number keys on the computer keyboard. The measured variables include categories completed (CC), total errors (TE), perseverative errors (PE), and nonperseverative errors (NRPE).
- ERP

##### Stimuli

Facial pictures are grayscale photographs selected from the native Chinese Facial Affective Picture System [16], and a total of 60 facial pictures are used as target faces (20 happy, 20 neutral, and 20 fear faces) with an equal number of males and females. Each picture was assessed for its valence and arousal on a 9-point scale with a large sample of Chinese participants in a previous survey. Each pair of facial stimuli will consist of a neutral picture and an emotional picture. Thus, we will have pairs of emotional-face stimuli consisting of 60 emotional faces, including 20 happy-neutral, 20 sad-neutral, and 20 fear-neutral pairs of facial stimuli.

##### Procedure

The experimental paradigm is a modified dot-probe paradigm. The task consists of 160 trials. A central fixation appears for 300 ms, and then replaced by a pair of emotional-face stimuli with different valences as a cue, which will last for 500 ms. After a short 200-ms interval, the dot probe is randomly presented for 150 ms as a target in either the left or right position of the fixation cross. The participant is asked to identify the spatial location of the “dot” and register the response with the forefinger pressing the button “1” or “4” on the reaction box as quickly as possible. An automatic interval of 2000 ms is programmed to receive the participants’ response; after a response is made or 2000 ms (whichever occurs first), a black screen is presented for 600 ms and followed by the subsequent trial [25].

##### ERP data

During the cognitive task, ERP data will be recorded by the NeuroScan ERP system (NeuroScan Company, American), which contains a 64-channel amplifier and a 64-electrode Ag/AgCL scalp cap. Each epoch following the emotional-face cue and target will mainly be analyzed by an off-line analysis using MATLAB 2013a (The Mathworks, Natick, USA) as well as its toolbox ERPLAB toolbox (http://www.erpinfo.org/erplab). The latency and average amplitudes of the three ERP components P100, N170, and P300 will be recorded and have been detected in various stages of attention processing.

##### Behavioral data

E-Prime software (Psychology Software Tools Inc.) will be applied to program the experimental procedure, record the accuracy of the responses, and the behavioral reaction times (RT) together with the ERP data.

### Safety evaluation

Adverse events (AEs) during the treatment will be recorded. AEs include the following: hematoma around the site of needling insertion points, local numbness, pain, and dizziness during treatment. If any AE occurs, the doctor will assess and provide corresponding treatment to the patient in accordance with the study protocol, and report the adverse events form in the case report form.

### Blinding assessment

The acupuncturists will ask participants to guess whether they received acupuncture treatment or sham acupuncture after the last treatment in week 4.

### Sample size

The study uses the HAMD-17 as the primary outcome measure for sample size calculation. The sample size was calculated using the following formula for the comparison of two samples:

$$ n=\frac{{\left({Z}_{1-\frac{a}{2}}+{Z}_{1-\beta}\right)}^2\times \left({\sigma}_1^2+{\sigma}_2^2\right)}{\delta^2} $$

In this study, a two-tailed test was chosen; σ_1_ and σ_1_ represent the difference in the change in HAMD-17 scores after treatment in the test group (acupuncture group) and placebo group (control group), respectively, and δ represents the difference between the two groups. Power Analysis and Sample Size software (PASS; version 11; NCSS Statistical Software, Kaysville, UT, USA) was used for sample size calculation. According to the results of a pilot study [26], σ_1_=9.50, σ_2_=7.42, δ=2.08, *α*=0.05, ${Z}_{1-\frac{a}{2}}$=1.96, *β*=0.1, and *Z*_1 − *β*_=1.28. The calculated sample size of each group was approximately 22 individuals to achieve a power of 0.90 at a significance level of 0.05. After accounting for an anticipated dropout rate of 20%, the total number of patients required for this trial was 56, with 28 in each group.

### Recruitment

The researchers will distribute trial information leaflets to the inpatient and outpatient clinics of the hospital. Posters will also be used of recruitment. Suitable participants will be encouraged to ask a question about the study and given time to decide if they wish to participate. Participants who are willing to participate will be asked to provide written consent.

### Allocation

#### Sequence generation

Participants will be randomly allocated to either the acupuncture group or the control group in a 1:1 ratio by simple randomization. The randomization schedule was calculated in the statistical software SPSS (IBM SPSS statistics version 22.0, USA) by a statistical expert from the Faculty of Medical Statistics and Epidemiology, The Second Affiliated Hospital of Guangzhou University of Chinese Medicine.

#### Allocation concealment mechanism and implementation

The allocation sequences are enclosed in sealed envelopes with a unique identification number. The envelope will be sent to an investigator in charge of the group assignments. The investigator will specify in the envelope who and when to open the randomly assigned envelope. The number will appear on all report forms to maintain participant confidentiality.

#### Blinding

The acupuncturists are not blinded to group allocation. Participants are blinded to their group allocation but are informed that they had an equal chance of allocation to the acupuncture or control group before study participation. The outcome assessor will also be blinded to group allocation. Adverse reactions are carefully observed, and emergency unblinding is required if serious adverse reactions occur. Unblinding will be performed at the end of the trial to perform statistical analysis.

#### Data collection methods

Before study initiation, all researchers and acupuncturists will participate in a 2-day training session including assessment and collection of outcome, baseline, and follow-up organized by the Secondary Medical College of Guangzhou University of Traditional Chinese Medicine. During the follow-up period, participants are requested to return to the clinic to receive assessment and will be given subsidies to cover transportation. If the participants could not complete the entire study, and lost to follow-up, these data will be included in the statistics and should be analyzed intentionally.

#### Data management

The data and safety monitoring committee are composed of independent clinicians and biostatisticians with rich experience in clinical trials. The ethics committee of the Secondary Medical College of Guangzhou University of Traditional Chinese Medicine will perform random audits to ensure compliance with relevant regulations and guidelines. Any changes of the research protocol will be communicated to both the ethics committee and registration center timely. The case report form (CRF) is a paper version and should be properly recorded by the investigator. One researcher will be responsible for collecting data at each stage of the study, while another researcher will input the data into the SPSS software. This process will be carried out under the supervision of project monitors. According to the terms of informed consent, unless the subject’s consent is obtained, all the subject’s personal information is confidential and will not be disclosed to the public. The sponsor should keep CRF for 5 years after the end of the research.

### Statistical analysis

Descriptive statistics will be used to describe demographic data of sample groups, while the normality of the distribution of each parameter will be checked by histogram and the Kolmogorov-Smirnov test. The continuous variables will be presented in mean and standard deviation (SD). A chi-squared test will be used to assess sample characteristics and gender. The data that conform to normal distribution will be analyzed by one-way analysis of variance, while the nonconforming data will be analyzed by Mann-Whitney *U* test. The latency and amplitude of the ERP data will be analyzed by mixed-effect model analysis, the Greenhouse-Geisser adjustment will be applied to adjust the degrees of freedom if the assumption of Mauchly’s test of sphericity is not significant. *P* < 0.05 will be taken as the standard for a significant difference. Data analysis will follow the intention-to-treat principle including participants who are randomly assigned. Missing data will be input with the last observation carried forward method.

### Ethics and dissemination

This study was approved by the ethics committee of the Secondary Medical College of Guangzhou University of Traditional Chinese Medicine, China, on 6 September 2019 (approval No. ZF2019-170-01). Protocol revision, adverse reaction reports, and annual review will be supervised by the ethics committee of the Secondary Medical College of Guangzhou University of Traditional Chinese Medicine. The research observers will explain the study to participants before they voluntarily sign the informed consent form at the beginning of the study. Participation in the study is voluntary and can be discontinued at any time. The decision not to participate will not affect the patient’s care. On the consent form, participants will be asked to grant permission for the research team to share relevant data with the researchers taking part in the research or regulatory authorities, where relevant. Participants will also be asked if they agree to the use of their data, should they choose to withdraw from the trial. This trial does not involve the collection of biological specimens for storage. Information provided by participants will only be shared with members of the research team. We will make every effort to keep the participants’ information confidential. All paper documents related to the study will be kept in a locked cabinet. All participants’ information will be kept strictly confidential. Clinical examination and acupuncture treatment related to this study are provided free of charge during the study period. All participants will be reimbursed for travel expenses to encourage adherence during the follow-up phase.

## Discussion

PSD is the most common emotional complication after stroke and characterized by sustained depression and loss of interest. It ranks first among the comorbidities of neurological diseases and depression and is related to poor functional outcomes and high recurrence and mortality. Antidepressants are commonly used in the treatment of depression, which can cause AEs and substance abuse. Therefore, it is very important to find other effective and safe treatments and explore the mechanism of antidepressants.

Acupuncture is one of the most popular nonpharmaceutical therapies for depression and has been increasingly recognized globally in recent years [27]. The literature shows that acupuncture has a positive effect on and improves depressive symptoms of PSD [11]. A recent meta-analysis demonstrated that acupuncture resulted in better outcomes compared with several groups of antidepressant medications without any obvious adverse reactions [13]. However, these trials did not consider placebo or sham acupuncture as control conditions to exclude the psychological effects of acupuncture.

Emotional and cognitive disorders are common psychological features underlying the depressive symptoms of MDD. ERP studies have shown that depressed patients have a tendency to overprocess negative stimulation, and the recognition accuracy of negative and positive information is decreased [28]. It has been found that those with MDD have difficulty shifting attention from negative information, which leads to persistent negative emotional rumination. We believe that this emotional cognitive processing deficit in PSD may manifest as an attentional bias to negative emotions. The existing studies on PSD are mainly based on responses to clinical scales to assess depressive symptoms but have not focused on the effects of acupuncture on cognition in those with PSD. However, the mechanisms of how acupuncture regulates attentional bias in those with PSD are still not clear. Therefore, this trial was designed as a randomized, placebo-controlled clinical trial to explore the clinical efficacy and safety of acupuncture therapy on PSD. Moreover, we will employ indices reflective of cognitive function as secondary outcomes, including the WCS T[29] and ER P[17] measures, with the expectation that they will provide reliable clinical evidence for acupuncture treatment that helps improve PSD.

There are also some limitations to the study. First, it is difficult to implement the double-blind approach as the acupuncturists could not be blinded, which may in performance bias. Second, due to the limitations of project funding and trial period, the follow-up period is only 1 month. Future trials should investigate the benefits of acupuncture treatment for PSD beyond 3 months. Moreover, different regions of cerebral infarction may lead to different degrees of depression and cognitive impairment. Future studies would be needed to investigate the relationship between different infarction regions and degrees of depression and cognitive function.

## Trial status

Trial registration: This study was registered with the Chinese Clinical Trial Registry on 27 October 2019 with the ID ChiCTR1900026948. The protocol version is 1.0, 1 July 2019. Patient recruitment was initiated on 1 December 2019 and is scheduled to be completed by the end of December 2021. If we should amend the protocol, we will communicate with the investigators, ethics committee, and trial registries.

## Supplementary Information

**Additional file 1.** Standard Protocol Items: Recommendations for Interventional Trials (SPIRIT) Checklist

## Data Availability

According to the terms of informed consent, unless the subject’s consent is obtained, all the subject’s personal information is confidential and will not be disclosed to the public. When necessary, the ethics committee or project funding department may consult the subject’s data. They will not use the subject’s information for other purposes or disclose it to other groups without permission.

## Abbreviations

PSD: Poststroke depression
WCST: Wisconsin Card Sorting Test
HAMD: Hamilton Depression Scale
MDD: Major depression disorder
ERP: Event-related potential
CT: Computed tomography
MRI: Magnetic resonance imaging
CC: Categories completed
TE: Total errors
PE: Perseverative errors
NRPE: Nonperseverative errors
RT: Reaction times
