# Correction: Efficacy of acupuncture on cognitive function in poststroke depression: study protocol for a randomized, placebo-controlled trial

**DOI:** 10.1186/s13063-025-08796-9

**Published:** 2025-04-18

**Authors:** Ling Chen, Yi Chen, Lihua Wu, Wen Fu, Luanmian Wu, Wenbin Fu

**Affiliations:** 1 Second Clinical College, Guangzhou University of Chinese Medicine, Guangzhou, China; 2 Guangzhou University of Chinese Medicine, Guangzhou, China; 3 Shenzhen Bao’an Research Center for Acupuncture and Moxibustion, Shenzhen, Guangdong Province China; 4 Sanming Project of Medicine in Shenzhen, Shenzhen, Guangdong Province China

**Correction: Trials 23, 85 (2022)**

**https://doi.org/10.1186/s13063-022-06011-7**

Following the publication of the original article [1], we were notified that a grant number was not included in the Funding section (because it was still unknown at the time of publication):

Grant Number:.ZY2022KY05

Funding Agency: Research Fund for Zhaoyang Talents of Guangdong Provincial Hospital of Chinese Medicine

The original article was corrected.
